# Development of a Child Articulation Screening Test Within Digital Therapeutics: Delphi Study

**DOI:** 10.2196/90110

**Published:** 2026-04-23

**Authors:** Jaewon Lee, Sejin Kwon, Jin Young Ko, Yulhyun Park, Jinju Lee, Ju Seok Ryu, Seo Yeon Yoon, Jee Hyun Suh

**Affiliations:** 1 Department of Rehabilitation Medicine, Seoul National University Bundang Hospital, 82, Gumi-ro 173 Beon-gil, Bundang-gu, Seongnam-Si, Gyeonggi-do, Republic of Korea, 82 31-787-7741; 2 Gyeonggi Provincial Medical Center, Anseong, Republic of Korea; 3 Gyeonggi Provincial Medical Center, Icheon, Republic of Korea; 4 Yonsei University College of Medicine, Seoul, Republic of Korea

**Keywords:** speech sound disorder, Delphi method, assessment tool, digital therapeutics, digital diagnostics, digital screening, pediatric screening, artificial intelligence, AI

## Abstract

**Background:**

Speech sound disorders are common in children and are associated with an increased risk of academic reading difficulties. The COVID-19 pandemic further highlighted the need for remote and digitalized assessment tools. In South Korea, standardized instruments such as the Urimal Test of Articulation and Phonation and Assessment of Phonology and Articulation for children are widely used but have limitations, including reliance on face-to-face evaluation, and the absence of automated scoring.

**Objective:**

This study aimed to develop and establish the content validity of an articulation assessment tool that can overcome these limitations and be integrated into digital therapeutics (DTx).

**Methods:**

A 3-round modified Delphi survey was conducted between July and September 2025 with 92% (23/25) of the invited experts, including 52.2% (12/23) physiatrists and 47.8% (11/23) speech-language pathologists, with a mean professional experience of 10.69 (SD 5.09) years. All participants (23/23, 100%) completed all rounds. Panelists evaluated the appropriateness of word lists, phonological environments, and scoring criteria. Quantitative analyses, including calculations of content validity ratio (CVR), content validity index (CVI), and median and IQR, were performed. Consensus thresholds were set at a CVR of ≥0.39, a CVI of ≥0.78, a median of ≥3.5, and an IQR of ≤1.0. Items were retained only when all 4 criteria were satisfied. While formal qualitative analysis was not performed, the research team internally reviewed and synthesized core keywords and themes from the experts’ open-ended responses to guide the refinement of items.

**Results:**

These findings were summarized into four key areas: (1) modernization of word stimuli, (2) expansion of phonological coverage, (3) refinement of scoring criteria to reduce ambiguity, and (4) enhancement of result interpretability through visualization. In round 2, a revised 35-word list was evaluated across 25 items, of which 20 (80%) met all consensus criteria. In total, 20% (5/25) of the items failed to meet at least one threshold, including phonological environment adequacy (CVR=0.48; CVI=0.74), scoring redundancy (CVR=0.13; CVI=0.57), usefulness of proportion of whole-word correctness or percentage of word proximity (CVR=0.39; CVI=0.70), contribution of mean phonological length (CVR=0.22; CVI=0.61), and usefulness of feature-based indexes (CVR=0.30; CVI=0.65; IQR 2). Items that reached consensus showed CVR values of 0.57 to 0.91, CVI values of 0.78 to 0.96, a median score of 4, and IQR values of 0 to 1. In round 3, all remaining items achieved consensus.

**Conclusions:**

This Delphi study developed a novel articulation assessment tool with robust content validity. This tool includes updated word stimuli, diverse analysis indexes, and visualization features, thereby enhancing its clinical utility and suitability for integration into artificial intelligence–based DTx. By standardizing and digitalizing articulation assessments, this tool has the potential to support personalized and accessible interventions for children with speech sound disorders.

## Introduction

Speech sound disorders (SSDs) represent one of the most prevalent communication disorders in childhood, typically manifesting as difficulty in accurate production or inappropriate use of speech sounds relative to developmental expectations for age [1]. Recent statistics suggest that approximately 24% of students with disabilities in US public schools are provided with services for speech or language disorders [2]. In South Korea, approximately 44.1% of children receiving speech therapy are diagnosed with SSDs, and approximately 9% of children aged 6 years present with articulation difficulties [3]. Children with SSDs are at increased risk of developing reading disabilities and other learning difficulties, resulting in significant implications for school readiness and overall academic achievement [4,5]. Therefore, early identification and intervention for SSDs are vital for reducing the risk of reading disabilities and promoting school readiness.

The COVID-19 pandemic had a profound global impact in 2020. Environmental factors, including potential effects of mask wearing, have been reported to negatively affect children’s cognitive, social, and language development [6,7]. However, effects related to the COVID-19 pandemic persist, continuing to negatively affect language development in children [7]. Telerehabilitation and digital therapeutics (DTx) reduce health care costs, save patients time, and improve accessibility [8]. Furthermore, following the COVID-19 pandemic, the use of telerehabilitation increased substantially, with reported rates increasing from 3.4% to 19.3% [9]. There is a growing shift toward remote and digital approaches for both assessment and intervention. In this context, appropriate tools for evaluating SSDs are needed to ensure standardized quality, allow for home-based administration, and minimize the need for in-person visits.

In South Korea, the Urimal Test of Articulation and Phonation (U-TAP) and Assessment of Phonology and Articulation for Children (APAC) are among the most frequently used standardized instruments for the evaluation of SSDs [3]. However, despite their widespread use, both tools present certain limitations. They rely heavily on face-to-face administration and perceptual assessment, lack automated or digitalized scoring systems, and have limited applicability in remote or home-based assessment settings. Moreover, normative data are constrained to specific age groups and linguistic contexts, posing challenges for their broader clinical use.

Beyond these logistical and systemic constraints, these conventional assessments also have technical shortcomings in their analytical frameworks. For instance, both the U-TAP and APAC are time-consuming [3], and the U-TAP does not distinguish between medial and final positions when evaluating codas, making it difficult to accurately identify coda errors [10]. In contrast, the APAC defines the omission of a medial coda or regressive assimilation of a following medial onset into the coda as typical medial simplification and the omission of a medial onset or progressive assimilation of a medial coda into the following onset as atypical medial simplification [10]. On the basis of this framework, the APAC analyzes coda errors separately for medial and final positions. However, it does not differentiate between omission and substitution errors of medial codas as they are all categorized under medial simplification, limiting the detailed analysis of medial coda errors [10].

While recent advances in artificial intelligence (AI), particularly large language models, have expanded the potential for DTx applications in speech-language pathology [11], existing research has predominantly focused on intervention efficacy rather than the development of linguistically valid and digitally adaptable assessment frameworks [11-13]. In particular, recent studies have attempted to apply AI technologies to the detection and assessment of SSDs. In the context of recent advances in digital health, several studies have explored AI-assisted or digital approaches in speech-language pathology. For example, previous work has developed automated SSD detection systems using deep learning models based on automatic speech recognition and audio classification, achieving moderate classification performance (eg, unweighted average recall of approximately 70%) [14]. However, these approaches primarily focus on classification tasks (eg, distinguishing children with typical development from those with SSD) and rely on metrics such as proportion of whole-word correctness (PWC) without providing detailed linguistic analysis of articulation error patterns. In addition, their performance is influenced by the accuracy of speech recognition models and predefined thresholds, which may limit interpretability and clinical applicability. Although such studies demonstrate the technical feasibility of AI-based SSD detection, clinically standardized and linguistically grounded assessment tools suitable for widespread diagnostic use remain under development. Therefore, there remains a lack of clinically grounded and digitally adaptable diagnostic frameworks that can accurately capture articulation error patterns while being suitable for digital implementation. In this regard, unlike previous AI-based SSD detection studies focused on classification accuracy, this study aimed to establish a linguistically structured and clinically interpretable assessment framework that can support detailed error pattern analysis and future integration into DTx systems.

Rapid AI-powered large language models have drawn significant attention for their potential use in DTx [11]. To fully realize this potential, it is essential to clarify how such interventions function, what factors affect their effectiveness, and how AI can be systematically integrated into DTx [11-13]. To develop AI-based DTx for children with SSDs effectively, it is essential to accurately identify each child’s specific error patterns and design algorithms to determine appropriate intervention strategies. The most fundamental step in this process is the precise evaluation of a child’s articulation error patterns.

A previous study reported that, while speech-language pathologists demonstrated high intrarater reliability in the evaluation of speech intelligibility, interrater reliability was only moderate to excellent, indicating variability across clinicians [15]. Thus, conventional speech-language pathologist–based language assessments are limited by variability in scoring and interpretation across clinicians. This limitation may contribute to inequitable and potentially discriminatory access to assessments and treatments across rural and urban regions.

Taken together, these limitations highlight the need for a novel articulation assessment tool that is not only clinically and linguistically robust but also specifically designed for digital and AI-based applications.

This study aimed to develop a new articulation development evaluation tool to overcome the technical and clinical limitations of the U-TAP and APAC, facilitate its transition to DTx, and verify its content validity and applicability through a Delphi study.

## Methods

### Study Design

The Delphi survey was conducted in 3 iterative rounds between July 2025 and September 2025. The number of rounds was predefined to a maximum of 3 to ensure a structured consensus-building process. We used a Delphi methodology to systematically gather expert consensus on digital assessment items for articulation and phonological disorders [16]. The Delphi technique collects expert consensus anonymously through structured questionnaires and iterative feedback [17]. It provides a systematic and objective approach for evaluating content that requires professional judgment and is widely recognized as a standard method for establishing the content validity of clinical assessment tools.

The initial questionnaire was developed by a multidisciplinary committee consisting of 3 physiatrists and 1 professor of speech-language pathology. The committee drafted the word list based on clinical experience, existing instruments (eg, U-TAP and APAC), and literature on word familiarity. Specifically, candidate words were selected to reflect age-appropriate vocabulary, phonetic balance across consonant positions, and clinical relevance for identifying common articulation error patterns. Relevant literature and existing standardized assessment tools were reviewed to ensure linguistic validity and familiarity for Korean-speaking children.

To minimize potential psychological biases, such as the bandwagon effect and interference effects; ensure anonymity; and enhance validity, we implemented a web-based modified Delphi process conducted via an online survey platform (Google Forms). All responses were collected anonymously, and participants were blinded to the identities of other panel members. Open- and closed-ended questions were used to facilitate consensus. In round 1, open- and closed-ended questions were used to evaluate the appropriateness of a word list and stimulus composition, diversity and usefulness of phonological environments, and validity of both scoring criteria. Round 2 served as an internal validation stage in which the results of round 1 were analyzed and restructured into closed-ended questions. Summarized feedback from round 1 was provided to participants in round 2 to support iterative consensus building. In round 3, additional feedback was incorporated to refine the items, and a final set was established. In rounds 2 and 3, a 5-point Likert scale was used supplemented with open-ended sections to allow participants to revise, add, or withdraw their opinions.

Between rounds, both quantitative and qualitative syntheses were performed. The feedback, including statistical summaries and qualitative syntheses, was provided uniformly to all participants to support consensus building. Although a formal qualitative analysis of open-ended responses was not performed, the research team internally reviewed and synthesized core keywords and themes to guide item refinement and establish priorities based on importance and appropriateness. Furthermore, even for items that reached statistical consensus, additional exploratory questions were included in subsequent rounds if the research team identified clinically significant issues requiring further expert deliberation. In addition, panelists were encouraged to consider the scalability and applicability of the assessment framework for future digital and AI-based implementation, including the use of structured, quantifiable scoring systems and standardized output formats that could support automated analysis.

### Participants

An expert panel was convened, with specialists identified through the official membership lists of the Korean Academy of Rehabilitation Medicine and the Korean Association of Speech-Language Pathologists. Initially, the research team considered including DTx developers; however, the scope was purposefully narrowed to specialists in rehabilitation medicine and speech-language pathology to focus on establishing the foundational clinical validity of the assessment tool. The panelists were chosen based on their expertise in articulation and phonological disorders and their experience in digital assessment or related fields.

According to previous studies, homogeneous panels typically require 15 to 30 participants, whereas heterogeneous panels may be sufficiently represented with 5 to 10 participants [18]. Given that our Delphi panel included 2 distinct professional groups—speech-language pathologists and physiatrists—the panel could be considered heterogeneous. Therefore, the inclusion of 23 experts in this study exceeded the recommended size for heterogeneous panels, ensuring both adequacy and diversity of perspectives.

The criteria for panel selection were as follows: (1) possession of a degree in the relevant specialty, (2) a minimum of 5 years of clinical or research experience, and (3) understanding of study objectives and methodology with consent to participate. Exclusion criteria were as follows: (1) less than 5 years of clinical or research experience, (2) willingness to participate without sufficient understanding of the study objectives or Delphi method, and (3) inability to continue participation due to withdrawal or incomplete responses during the survey rounds. These inclusion and exclusion criteria were established a priori. A total of 25 potential experts were initially invited via email. Of these 25 experts, 23 (92%) agreed to participate, whereas 2 (8%) declined due to scheduling conflicts. No participants were excluded after enrollment, and all 23 experts completed all Delphi rounds and were included in the final analysis.

### Ethical Considerations

This study was approved by the Institutional Review Board of Seoul National University Bundang Hospital (B-2507-987-301). All the participating experts were provided with information regarding the purpose of the study, expected time commitment, eligibility criteria, and importance of completing all rounds of the survey. Participation in the Delphi process was voluntary, and informed consent was obtained prior to involvement. Participants received monetary compensation of US $100 for their time and participation in the study. The compensation was determined to be appropriate and not coercive. To ensure anonymity, the survey was conducted online, and each expert was assigned a unique identification code. This procedure allowed responses to remain confidential while maintaining the integrity of the iterative Delphi process. All the responses were collected anonymously to protect participants’ confidentiality.

### Data Analysis

All responses were analyzed using equal weights. Predefined criteria for consensus were established prior to data collection to guide interpretation and agreement thresholds. To ensure content validity, basic descriptive statistics (mean, SD, and median), content validity ratio (CVR), and consensus indexes were examined and analyzed. CVR was calculated to assess the essentiality of each word item based on the number of panelists rating an item as “essential.” Following the Lawshe formula, the minimum acceptable CVR value for 23 panelists was set at 0.37 [19]. Additionally, the content validity index (CVI) was calculated from a percentage of panelists giving each item a rating of 3 or 4 on a 5-point Likert scale, with a CVI of 0.78 or higher indicating high content validity [20].

Consensus levels were evaluated using the median and IQR. A median score of 3.5 or higher was regarded as positive consensus, whereas an IQR of 1.0 or lower indicated a high level of agreement among panelists [21].

Items were retained if they met all 4 criteria: CVR of 0.39 or higher, CVI of 0.78 or higher, median of 3.5 or higher, and IQR of 1.0 or lower. Items that met CVR and CVI thresholds but failed to reach consensus (median or IQR) were revised and re-evaluated in a subsequent round, whereas items that did not meet the CVR or CVI thresholds were either modified or eliminated.

Although a formal qualitative analysis was not conducted, open-ended responses were reviewed using a structured descriptive approach. Two investigators independently examined the responses to identify recurring themes and clinically relevant insights. Discrepancies were resolved through discussion, and the synthesized findings were used to refine questionnaire items and inform subsequent Delphi rounds.

## Results

### Overview

**Figure 1.**
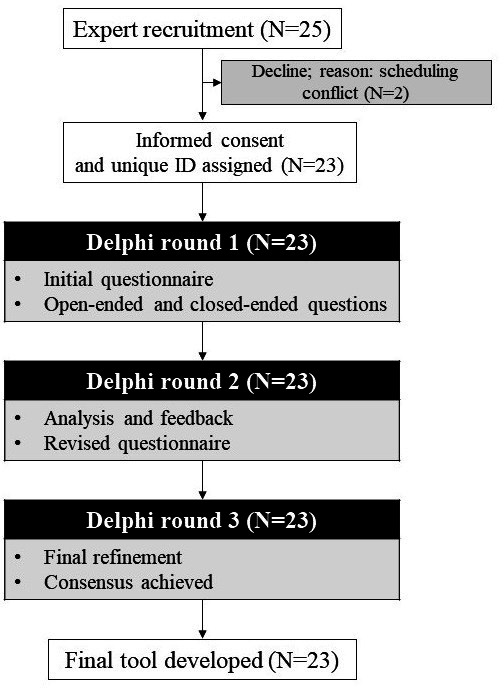
Flowchart of the 3-round Delphi process used to develop the articulation assessment tool. Of the 25 recruited experts, 23 (92%) participated in all rounds. The process included an initial assessment (round 1), feedback and revision (round 2), and final consensus (round 3), resulting in the final tool.

This study aimed to develop a diagnostic tool for children with SSD to support the creation of an AI-based DTx instrument. The three main objectives were to (1) construct a word list and diagnostic tool for children with SSD, (2) evaluate the content validity of the developed tool, and (3) establish diagnostic parameters for differentiating SSD using a Delphi survey process. The findings of the 3 study rounds (Figure 1 shows a flowchart of participation) are described in the following sections.

### Round 1

The first round of the Delphi survey was completed by all 23 experts who initially agreed to participate, achieving a 100% (23/23) response rate for this stage. The panel was asked open-ended and multiple-choice (5-point scale) questions regarding the appropriateness of the 26 initial words, diversity of phonological environments, and validity of the scoring criteria. On the basis of the comprehensive feedback from the 23 experts, the panel emphasized the need to include diphthong words, exclude words with potential dialectal variations (eg, *jajangmyeon* [black bean noodles]), and remove words unfamiliar to modern urban children (eg, *gulttuk* [chimney] and *radio*). They also highlighted the necessity of expanding and supplementing words containing fricative and affricate codes as well as increasing the overall number of test words.

Furthermore, the experts recommended incorporating listeners’ subjective ratings of speech clarity, clarifying the scoring criteria for ambiguous productions, improving the scoring system, and presenting the results using graphical visualization. These qualitative insights, synthesized from the open-ended responses, provided the foundational rationale for the modifications implemented in the subsequent round. Notably, several of these refinements, such as the development of clearer scoring criteria and graphical visualization of results, were aligned with the need to generate structured and interpretable data that may be suitable for future AI-based analysis.

### Round 2

Following the synthesis of results from the 23 panel members who responded to the round 1 Delphi survey, words from an initial 26-item list that were deemed inappropriate for modern urban children or had the potential to be dialectal were excluded (Table 1).

**Table 1.** Word list for articulation assessment developed through a 3-round Delphi survey among experts in pediatric rehabilitation and speech-language pathology (n=35 words).

Target word in Korean	Meaning	IPA^a^ transcription
타조	Ostrich	[tʰa̠.d͡ʑo̞]
냄비	Pot	[nɛm.bi]
눈썹	Eyebrow	[nun.s͈ʌp̚]
딸기	Strawberry	[t͈a̠l.ɡi]
국자	Ladle	[kuk̚.t͡ɕ͈a̠]
다리	Leg and bridge	[ta̠.ɾi]
자동차	Car	[t͡ɕa̠.do̞ŋ.t͡ɕʰa̠]
씨앗	Seed	[ɕ͈i.a̠t̚]
바나나	Banana	[pa̠.na̠.na̠]
레몬	Lemon	[ɾe̞.mo̞n]
코끼리	Elephant	[kʰo̞.k͈i.ɾi]
곰	Bear	[ko̞m]
사탕	Candy	[sa̠.tʰa̠ŋ]
버섯	Mushroom	[pʌ.sʌt̚]
펭귄	Penguin	[pʰe̞ŋ.ɡɥin]
마이크	Microphone	[ma̠.i.kʰɯ]
연필	Pencil	[jʌn.pʰil]
까치	Magpie	[k͈a̠.t͡ɕʰi]
빵	Bread	[p͈a̠ŋ]
아빠	Dad	[a̠p͈a̠]
책상	Desk	[t͡ɕʰɛk̚.s͈a̠ŋ]
찌개	Stew	[t͡ɕ͈i.ɡɛ]
오뚝이	Roly-poly toy	[o̞.t͈u.ɡi]
하마	Hippopotamus	[ha̠.ma̠]
구급차	Ambulance	[ku.ɡɯp̚.t͡ɕʰa̠]
수박	Watermelon	[su.ba̠k̚]
과자	Snack	[kwa̠.d͡ʑa̠]
돼지	Pig	[twe̞.d͡ʑi]
요거트	Yogurt	[jo̞.ɡʌ.tʰɯ]
귀	Ear	[kɥi]
여우	Fox	[jʌ.u]
우유	Milk	[u.ju]
야구	Baseball	[ja̠.ɡu]
원숭이	Monkey	[wʌn.su.ŋi]
의자	Chair	[ɰi.d͡ʑa̠]

a IPA: International Phonetic Alphabet.

These were replaced with alternative items, along with additional words representing diverse articulatory environments (eg, *remon* [레몬], meaning “lemon,” *ottugi* [오뚝이], meaning “roly-poly toy,” and *jjigae* [찌개], meaning “stew”). Furthermore, words containing diphthongs (eg, *yagu* [야구], meaning “baseball,” *uija* [의자], meaning “chair,” *wonsungi* [원숭이], meaning “monkey,” *yogeoteu* [요거트], meaning “yogurt,” *dwaeji* [돼지], meaning “pig,” and *gwaja* [과자], meaning “snack” or “cracker”) were incorporated, resulting in a final test list of 35 words. To facilitate a clearer interpretation of speech outcomes by both caregivers and clinicians, visual graphs were integrated into the scoring results to display consonant and vowel accuracies (Table 2).

**Table 2.** Consensus articulation assessment measures and scoring system established through a 3-round Delphi survey.

Articulation outcome category and subcategory	Denominator	Unit used for scoring
Accuracy		
Consonant accuracy (PCC^a^)	48	Percentage
Revised consonant accuracy (PCC-revised)	48	Percentage
Total consonant accuracy (total PCC)	48	Percentage
Total revised consonant accuracy (total PCC-revised)	48	Percentage
Vowel accuracy (PVC^b^)	14	Percentage
Diphthong accuracy	0	Percentage
Word accuracy (PWC^c^)	26	Percentage
PMLU^d^	26	Percentage
Articulation manner		
Plosive	23	Percentage
Fricative	5	Percentage
Affricate	6	Percentage
Nasal	10	Percentage
Liquid	4	Percentage
Articulation place		
Bilabial	12	Percentage
Alveolar	19	Percentage
Palatal	6	Percentage
Velar	10	Percentage
Glottal	1	Percentage
Phonological features		
Continuancy	6	Percentage
Delayed release	6	Percentage
Laterality	3	Percentage
Coronal	23	Percentage
Anterior	27	Percentage
Tenseness	18	Percentage
Aspiration	8	Percentage
High tongue	14	Percentage
Back tongue	10	Percentage
Roundedness	3	Percentage
High	6	Percentage
Low	1	Percentage
Back	7	Percentage
Error patterns—substitution		
Plosivization	21	Percentage
Fricativization	37	Percentage
Affricativization	37	Percentage
Nasalization	36	Percentage
Liquidization	40	Percentage
Bilabialization	33	Percentage
Alveolarization	26	Percentage
Palatalization	37	Percentage
Velarization	35	Percentage
Glottalization	41	Percentage
Tense consonantization	34	Percentage
Aspirated consonantization	33	Percentage
Error patterns—assimilation		
Plosive assimilation	16	Percentage
Fricative assimilation	5	Percentage
Affricate assimilation	8	Percentage
Nasal assimilation	12	Percentage
Liquid assimilation	9	Percentage
Bilabial assimilation	11	Percentage
Alveolar assimilation	13	Percentage
Palatal assimilation	8	Percentage
Velar assimilation	14	Percentage
Glottal assimilation	2	Percentage
Tense assimilation	9	Percentage
Aspirated assimilation	9	Percentage

a PCC: percentage of consonants correct.

b PVC: percentage of vowels correct.

c PWC: proportion of whole-word correctness.

d PMLU: phonological mean length of utterance.

Subsequently, the round 2 Delphi survey used a 5-point Likert scale. This survey was conducted with the same 23 experts who completed the previous round, maintaining a 100% (23/23) retention rate for this round. The survey comprised 7 items that evaluated the appropriateness of the word list and stimulus composition, 5 items assessing the diversity and adequacy of phonological environments within the word list, and 13 items examining the validity of the scoring criteria (Table S2 in Multimedia Appendix 1).

In the second-round Delphi survey, most items demonstrated strong expert agreement, meeting the predetermined consensus thresholds of CVR of 0.39 or higher, CVI of 0.78 or higher, median of 3.5 or higher, and IQR of 1.0 or lower. These results indicate that most items of the word list, phonological environments, and scoring criteria were assessed to be appropriate and valid for clinical applications.

However, no consensus was reached regarding 5 items. Specifically, experts did not agree on whether (1) the phonological environments adequately reflected common child speech errors, (2) the scoring rubric was excessively detailed or redundant, (3) analyses using PWC or percentage of word proximity were useful for setting treatment goals, (4) the mean phonological length contributed effectively to the evaluation of phonological complexity, and (5) feature-based indexes provided practical benefits for error pattern analysis (Table 3). The specific results for each round, including items that achieved consensus and those that did not, are presented in Table 3 to provide a transparent overview of the refinement process.

**Table 3.** Outcomes of the second-round Delphi survey evaluating the appropriateness of the word list, phonological environments, and scoring criteria for articulation assessment.

Category and survey question	CVR^a^	CVI^b^	Median (IQR)	Agreement (proportion, %)
Word list and stimulus appropriateness				
Word list matches assessment purpose	0.83	0.91	4 (0)	1
Word list appropriate for the ages of 2‐18 y; familiar vocabulary	0.65	0.83	4 (0)	1
Words difficult to visualize or confusing	0.83	0.91	4 (0)	1
Presentation order appropriate for flow and difficulty	0.83	0.91	4 (0)	1
Word list appropriate for frequency and exposure by age	0.91	0.96	4 (1)	0.75
Word length appropriate	0.83	0.91	4 (1)	0.75
Factors to prioritize for engaging stimulus words	0.83	0.91	4 (0.5)	0.88
Phonological environments				
Adequate coverage of consonant positions, places, manners, and features	0.83	0.91	4 (0)	1
Selection of target vowels valid	0.74	0.87	4 (1)	0.75
Environments reflect common child speech errors	0.48	0.74^c^	4 (0.5)	0.88
Exclude dialectal or regional words	0.74	0.87	4 (0)	1
Option for diphthongs due to increased task difficulty or vocabulary load	0.74	0.87	4 (1)	0.8
Scoring criteria				
Understanding of indexes (PCC^d^, PMLU^e^, PWP^f^, and PVC^g^)	0.65	0.83	4 (0)	1
Usefulness of indexes in clinical practice	0.65	0.83	4 (1)	0.75
Validity of consonant error classification	0.91	0.96	4 (0)	1
Clarity of scoring criteria (place, manner, phonation, and features)	0.91	0.96	4 (1)	0.75
Appropriateness of scoring method	0.57	0.78	4 (0)	1
Sufficiency of indexes for clinical interpretation	0.57	0.78	4 (0.5)	0.88
Scoring rubric excessively detailed or redundant	0.13^c^	0.57^c^	4 (1)	0.75
Appropriateness for caregiver explanation	0.65	0.83	4 (1)	0.75
Usefulness of PWC^h^ and PWP for goal setting	0.39	0.70^c^	4 (1)	0.75
Contribution of mean phonological length to complexity	0.22^c^	0.61^c^	4 (1)	0.75
Usefulness of accuracy by place and manner	0.91	0.96	4 (1)	0.75
Contribution of feature-based indexes to error analysis	0.30^c^	0.65^c^	4 (2)^c^	0.5^c^
Need for comprehensive report format	0.57	0.78	4 (1)	0.75

a CVR: content validity ratio.

b CVI: content validity index.

c Did not meet the predefined validity criteria.

d PCC: percentage of consonants correct.

e PMLU: phonological mean length of utterance.

f PWP: percentage of word proximity.

g PVC: percentage of vowels correct.

h PWC: proportion of whole-word correctness.

### Round 3

The third-round Delphi survey was conducted with the same 23 experts, maintaining a 100% (23/23) retention rate across all 3 rounds of the study. This final round was developed based on the results of the second round, incorporating modifications that were refined through the internal synthesis of expert keywords and thematic feedback from previous open-ended responses. Following this process, percentage of word proximity was removed, and the measurement of mean phonological length was modified to a supplementary index.

The five items addressed in the third-round Delphi survey were as follows: (1) word-level phonological environments adequately reflect contexts in which children’s speech errors commonly occur, (2) the scoring rubric is excessively detailed or redundant, (3) analyses using PWC are useful for establishing treatment goals, (4) the measurement of mean phonological length can serve as a supplementary indicator, and (5) consonant feature analysis contributes to the identification of error patterns. As a result, all 5 items reached consensus, meeting the thresholds of CVR of 0.39 or higher, CVI of 0.78 or higher, median of 3.5 or higher, and IQR of 1.0 or lower. This achieved the final expert consensus required for the development of the assessment tool.

## Discussion

### Principal Findings

In this study, we developed and validated stimulus words and an interpretation framework for articulation assessment in children with SSD using a 3-round Delphi process [22]. The resulting tool comprises 35 words that achieved expert consensus for developmental appropriateness and comprehensibility and incorporates updated stimuli, diverse analytical indexes, and visualization features to enhance clinical applicability and potential integration into AI-based DTx.

Compared with conventional standardized assessments such as the U-TAP and APAC, this tool introduces several important methodological and practical differences. First, while existing tools primarily rely on face-to-face administration and perceptual evaluation, our tool was designed with a structured and digitally adaptable framework, facilitating future integration into AI-based systems. Second, unlike the U-TAP, which does not distinguish between medial and final coda positions, this framework allows for more detailed positional analysis of phonological errors [10]. Third, although the APAC distinguishes positional coda errors, it does not differentiate between omission and substitution errors in medial codas; in contrast, our framework enables more granular classification of error patterns through structured indexes and feature-based analysis [10]. Furthermore, it enables the generation of diverse articulation analysis outcomes that can serve as a foundation for AI-based DTx. By standardizing and digitalizing articulation assessments, this tool has the potential to support personalized and accessible interventions for children with SSDs. Importantly, the structured and multidimensional output generated by this framework may facilitate its use as input data for future AI-based diagnostic or decision support systems. These findings extend prior AI-based SSD research by moving beyond classification-focused approaches toward a clinically interpretable and linguistically structured assessment framework. While previous studies have demonstrated the feasibility of automated detection, they have been limited in capturing detailed articulation error patterns and supporting clinical decision-making [14]. In contrast, this study emphasizes structured error analysis and interpretability, which are essential for clinical applicability and future integration into DTx systems.

These findings should be interpreted in relation to the gaps identified in the Introduction section. Specifically, this study addresses the lack of standardized and digitally adaptable assessment frameworks by establishing a structured scoring system and expanding phonological coverage. In addition, the incorporation of visualization features and multiple analytical indexes may contribute to improved interpretability and potential reduction in ambiguity in clinical assessment. However, some previously identified limitations remain unresolved [15]. In particular, interrater variability in perceptual judgment was not directly addressed, and automated or AI-based scoring systems were not implemented or validated in this study. Therefore, this work should be considered a foundational step toward digital assessment rather than a fully operational digital diagnostic system. In particular, while this study establishes a structured and AI-compatible assessment framework, further work is required to implement and validate automated or AI-driven diagnostic models based on this framework.

Compared with conventional assessment tools, which are often limited to static and paper-based formats, this tool provides a flexible and extensible digital framework. This aligns with recent trends in digital health and AI-assisted diagnostics, where standardized input and scalable data structures are essential for model training and clinical deployment.

This study holds significant value in establishing clinical content validity and a systematic derivation methodology, addressing the limitations of existing analog tools and the challenges of digital transition. To ensure that this tool functions as a reliable diagnostic system within a DTx environment, several directions for future research are proposed. First, the clinical reliability and validity of the word list and assessment system should be empirically verified through correlation analyses using existing standardized instruments (eg, U-TAP and APAC). In addition, large-scale normative data across different ages and genders should be collected to establish percentile-based reference values and objective diagnostic criteria for distinguishing typical from delayed development. Second, optimization studies addressing technical variables in real-world digital environments are required. These include evaluating the effects of environmental factors, such as background noise and device-related variability (eg, microphone performance), on the accuracy of AI-based automated scoring. Furthermore, systematic investigations are needed to establish technical guidelines that ensure consistent and reliable performance in home-based settings. The relationship between the linguistic properties of the target words and speech recognition performance should also be examined to support robust implementation. Finally, future studies should incorporate user interface and user experience design considerations. In particular, the cognitive load imposed on children in screen-based assessment environments should be carefully evaluated. Factors such as visual layout, stimulus presentation timing, and interaction design may influence attention, comprehension, and response accuracy. Accordingly, interdisciplinary collaboration with user interface and user experience specialists and educational technology developers will be essential to optimize usability and real-world applicability in DTx systems.

In addition, the interpretation of visualized assessment results should be approached with caution. Graphical outputs are intended to support understanding rather than serve as stand-alone diagnostic indicators, and clinical interpretation by qualified professionals remains essential to avoid potential misinterpretation. Furthermore, given that children’s voice data constitute sensitive personal information, future implementation of this tool within DTx environments must incorporate robust data security measures and ethical management frameworks. This includes ensuring data privacy, secure storage, and compliance with relevant regulatory standards.

### Limitations

This study has several limitations. First, although the Delphi method ensures content validity through expert consensus, it does not provide empirical validation of clinical reliability or diagnostic accuracy. Second, the panel consisted of experts from 2 professional groups, which, while appropriate for the study aims, may limit the generalizability of the findings to other disciplines or international contexts. Third, formal qualitative analysis of open-ended responses was not conducted, which may have limited the depth of thematic interpretation.

### Conclusions

Overall, this study provides a foundational framework for the development of digitally enabled articulation assessment tools and highlights the potential for integrating clinically validated instruments into AI-based therapeutic systems.

Future studies should verify the noninferiority of the newly developed articulation assessment tool by comparing its outcomes, such as consonant and vowel accuracy, with those of standardized instruments (eg, U-TAP). Additionally, the results of evaluations by speech-language pathologists should be compared with those generated by an AI-based assessment tool within the application to further establish its clinical validity. Furthermore, integration of this tool into DTx should be followed by clinical trials to evaluate its real-world effectiveness.

## Supplementary material

10.2196/90110 Multimedia Appendix 1 Round 1-3 Delphi questionnaire.

10.2196/90110 Checklist 1 DELPHISTAR checklist.

## Abbreviations

AI: artificial intelligence
APAC: Assessment of Phonology and Articulation for Children
CVI: content validity index
CVR: content validity ratio
DELPHISTAR: Delphi Studies in Social and Health Sciences–Recommendations for a Standardized Reporting
DTx: digital therapeutics
PWC: proportion of whole-word correctness
SSD: speech sound disorder
U-TAP: Urimal Test of Articulation and Phonation
