# Supplementary material for: Development of a Child Articulation Screening Test Within Digital Therapeutics: Delphi Study
Source: JMIR Form Res. 2026 Apr 23;10:e90110. doi: 10.2196/90110 (PMC13105429; doi:10.2196/90110)
Supplement: Multimedia Appendix 1 [file formative-v10-e90110-s001.docx]

**Supplementary 1.** Round 1 Delphi questionnaire

| **Section Ⅰ. General and Demographic Characteristics**   \| 1. What is your primary clinical specialty? (Multiple choice)  Pediatrics (Infants/Toddlers/School-age children) Adolescents Adults  Other (please specify: __________)  2. Have you ever used a digital-based language assessment system? (Single choice)  Here, a digital-based language assessment system refers to an application or online platform designed to assess a child’s language development status.  Yes No  3. If you have used a digital language assessment tool (e.g., app or web-based), what aspects did you find useful, and what improvements do you think are needed? (Open-ended)  (__________________________________________________________________________________) \| \| --- \|   **Section Ⅱ. Appropriateness of Word List and Stimulus Word Composition**   \| **1)** Do you think the current word list included in the articulation assessment tool aligns with the purpose of the assessment (e.g., phoneme coverage, diversity of articulation contexts)? Please describe it freely. (Open-ended) \| \| --- \| \| **2)** If there are articulation contexts that you believe are missing from the presented word list (e.g., voiced fricatives, complex final consonants), please specify them. (Open-ended) \| \| **3)** Do you think the current word list is appropriate for covering children aged 2 to 18 years, and that it is composed of vocabulary familiar to children in clinical settings? Please describe it freely. (Open-ended) \| \| **4)** If there are words that are difficult to visualize or may cause confusion, please list them with examples. (Open-ended) \| \| **5)** Do you think the order in which the words are presented is appropriate in terms of assessment flow or difficulty level? (Open-ended) \| \| **6)** Do you think the word list is appropriately composed considering age-appropriate word frequency and language exposure?   \| 1  (Strongly disagree) \| 2 \| 3 \| 4 \| 5  (Strongly agree) \| \| --- \| --- \| --- \| --- \| --- \|   ※ If there are words that are too unfamiliar or too uncommon and may interfere with the assessment, please specify them. (Open-ended) \| \| **7)** Do you think the composition of word length (monosyllabic words, disyllabic words, and words with three or more syllables) is appropriate?   \| 1  (Strongly disagree) \| 2 \| 3 \| 4 \| 5  (Strongly agree) \| \| --- \| --- \| --- \| --- \| --- \| \| \| **8)** In selecting stimulus words to engage children’s interest, what factors do you think should be prioritized? (Open-ended) \|   **Section Ⅲ. Diversity and Necessity of Phonological Context Composition**   \| **1)** Do you think the current word list includes a balanced representation of target consonant positions (word-initial, word-medial, word-final), as well as various places and manners of articulation, and features (e.g., plosives, fricatives, labials)?   \| 1  (Strongly disagree) \| 2 \| 3 \| 4 \| 5  (Strongly agree) \| \| --- \| --- \| --- \| --- \| --- \| \| \| --- \| --- \| --- \| --- \| --- \| --- \| \| **2)** Do you consider the selection of target vowels in the word list to be appropriate? Additionally, do you think including diphthongs, in addition to monophthongs, would be clinically useful?   \| 1  (Strongly disagree) \| 2 \| 3 \| 4 \| 5  (Strongly agree) \| \| --- \| --- \| --- \| --- \| --- \|     ※ Please feel free to share your opinion or provide examples of diphthongs that should be included.  (Open-ended) \| \| **3)** Do you think the target phonological contexts presented in the table adequately reflect contexts in which errors are frequently observed in actual child speech production?   \| 1  (Strongly disagree) \| 2 \| 3 \| 4 \| 5  (Strongly agree) \| \| --- \| --- \| --- \| --- \| --- \| \| \| **4)** Do you think words that may cause errors in speakers using dialects or regional varieties should be excluded? (Open-ended) \| \| **5)** Please freely provide any additional suggestions for enhancing the diversity of phonological contexts. (Open-ended) \|   **Section Ⅳ. Validity of Scoring Criteria and Feature-Based Assessment Method**   \| **1)** Do you clearly understand the distinctions among various indices for articulation accuracy (e.g., Percentage of Consonants Correct; PCC, Phonological Mean Length of Utterance; PMLU, Proportion of Whole-word Proximity; PWP, Percentage of Vowels Correct; PVC)? (Open-ended) \| \| --- \| \| **2)** Do you think these indices help capture children’s articulation characteristics more precisely in clinical practice?   \| 1  (Not helpful at all) \| 2 \| 3 \| 4 \| 5  (Very helpful) \| \| --- \| --- \| --- \| --- \| --- \|   ※ Please specify any indices you find particularly useful in practice or those that require improvement. (Open-ended) \| \| **3)** Do you think the current classification of consonant error types (e.g., phonological substitution errors, assimilation errors) by detailed articulation features is valid as an assessment tool?   \| 1  (Not valid at all) \| 2 \| 3 \| 4 \| 5  (Very valid) \| \| --- \| --- \| --- \| --- \| --- \|   ※ If you think it is invalid, please describe the reasons or elements that require modification. (Open-ended). \| \| **4)** Do you think the classification criteria in the scoring table (place of articulation, manner of articulation, voicing, distinctive features, etc.) are clinically clear and distinguishable?   \| 1  (Very unclear) \| 2 \| 3 \| 4 \| 5  (Very clear) \| \| --- \| --- \| --- \| --- \| --- \|   ※ If there are overlapping or confusing categories, please provide examples. (Open-ended) \| \| **5)** Do you think the current scoring calculation method (e.g., denominator based on total number of items, ratio representation) is appropriate for respondents or clinicians to understand and apply?   \| 1  (Very inappropriate) \| 2 \| 3 \| 4 \| 5  (Very appropriate) \| \| --- \| --- \| --- \| --- \| --- \|   ※ Please describe any areas in which the scoring method could be improved. (Open-ended) \| \| **6)** Do the current scoring indices (e.g., PCC, PMLU, PWP, PVC) provide sufficient information for clinically interpreting children’s articulation and phonological abilities?   \| 1  (Strongly disagree) \| 2 \| 3 \| 4 \| 5  (Strongly agree) \| \| --- \| --- \| --- \| --- \| --- \|   ※ Please specify any indices that are difficult to interpret or require interpretation guidelines. (Open-ended) \| \| **7)** Do you think the classifications in the current scoring criteria (features, place of articulation, manner of articulation, error types, etc.) are overly subdivided or contain redundancies?   \| 1  (Strongly disagree) \| 2 \| 3 \| 4 \| 5  (Strongly agree) \| \| --- \| --- \| --- \| --- \| --- \|   ※ If there are overlapping or confusing categories, please provide examples. (Open-ended) \| \| **8)** Do you think this scoring index system is appropriate for explaining changes in articulation abilities before and after treatment to parents or caregivers?   \| 1  (Very inappropriate) \| 2 \| 3 \| 4 \| 5  (Very appropriate) \| \| --- \| --- \| --- \| --- \| --- \|   ※ Please specify any indices that need to be visualized or simplified for caregiver explanation. (Open-ended) \| \| **9)** If you believe there are aspects that should be improved to increase the discriminant validity of the assessment, please describe them. (Open-ended) \| \| **10)** Do you think using PWC and PWP analyses is helpful in setting actual treatment goals?   \| 1  (Strongly disagree) \| 2 \| 3 \| 4 \| 5  (Strongly agree) \| \| --- \| --- \| --- \| --- \| --- \|   ※ Please provide any additional comments. (Open-ended) \| \| **11)** Do you think the PMLU scale effectively contributes to evaluating phonological complexity in children?   \| 1  (Strongly disagree) \| 2 \| 3 \| 4 \| 5  (Strongly agree) \| \| --- \| --- \| --- \| --- \| --- \| \| \| **12)** Do you think providing accuracy analysis results by manner of articulation (plosives, fricatives, etc.) and place of articulation (bilabials, velars, etc.) is useful for clinical interpretation?   \| 1  (Strongly disagree) \| 2 \| 3 \| 4 \| 5  (Strongly agree) \| \| --- \| --- \| --- \| --- \| --- \| \| \| **13)** Do you think introducing assessment indices based on distinctive features (continuancy, stridency, anteriority, etc.) can meaningfully contribute to error pattern analysis?   \| 1  (Strongly disagree) \| 2 \| 3 \| 4 \| 5  (Strongly agree) \| \| --- \| --- \| --- \| --- \| --- \| \| \| **14)** Do you think it is necessary to provide a comprehensive report format that includes multiple analysis indices (PCC, PWC, feature-based indices, etc.)?   \| 1  (Strongly disagree) \| 2 \| 3 \| 4 \| 5  (Strongly agree) \| \| --- \| --- \| --- \| --- \| --- \| \| |
| --- | --- | --- | --- | --- | --- | --- | --- | --- | --- | --- | --- | --- | --- | --- | --- | --- | --- | --- | --- | --- | --- | --- | --- | --- | --- | --- | --- | --- | --- | --- | --- | --- | --- | --- | --- | --- | --- | --- | --- | --- | --- | --- | --- | --- | --- | --- | --- | --- | --- | --- | --- | --- | --- | --- | --- | --- | --- | --- | --- | --- | --- | --- | --- | --- | --- | --- | --- | --- | --- | --- | --- | --- | --- | --- | --- | --- | --- | --- | --- | --- | --- | --- | --- | --- | --- | --- | --- | --- | --- | --- | --- | --- | --- | --- | --- | --- | --- | --- | --- | --- | --- | --- | --- | --- | --- | --- | --- | --- | --- | --- | --- | --- | --- |

**Supplementary Table 2.** Round 2 Delphi questionnaire

| **Section Ⅱ. Appropriateness of Word List and Stimulus Word Composition**   \| **1)** Do you think the current word list included in the articulation assessment tool aligns with the purpose of the assessment (e.g., phoneme coverage, diversity of articulation contexts)?   \| 1  (Strongly disagree) \| 2 \| 3 \| 4 \| 5  (Strongly agree) \| \| --- \| --- \| --- \| --- \| --- \|     ※ **Expert opinions were incorporated regarding the lack of diphthongs, the underrepresentation of certain phonemes, and the distinction between medial and final coda consonants** \| \| --- \| --- \| --- \| --- \| --- \| --- \| \| **2)** If there are articulation contexts that you believe are missing from the presented word list (e.g., voiced fricatives, complex final consonants), please specify them. (Open-ended)  **※ Expert opinions were incorporated regarding the lack of diphthongs, the underrepresentation of certain phonemes, and the evaluation of coda consonants by positional context** \| \| **3)** Do you think the current word list is appropriate for covering children aged 2 to 18 years, and that it is composed of vocabulary familiar to children in clinical settings?   \| 1  (Strongly disagree) \| 2 \| 3 \| 4 \| 5  (Strongly agree) \| \| --- \| --- \| --- \| --- \| --- \|   **※ Certain words unfamiliar to children were substituted with more appropriate alternatives (ex. Radio→Lemon)** \| \| **4)** In your opinion, is the revised word list suitable for visualization and readily comprehensible to children?   \| 1  (Strongly disagree) \| 2 \| 3 \| 4 \| 5  (Strongly agree) \| \| --- \| --- \| --- \| --- \| --- \|   **※ Terms that were difficult to visualize, or likely to be challenging for children to comprehend even when visualized, were revised** \| \| **5)** Do you think the order in which the words are presented is appropriate in terms of assessment flow or difficulty level?   \| 1  (Strongly disagree) \| 2 \| 3 \| 4 \| 5  (Strongly agree) \| \| --- \| --- \| --- \| --- \| --- \|   **※ Suggestions, including the placement of easier items at the beginning, were taken into account** \| \| **6)** Do you think the word list is appropriately composed considering age-appropriate word frequency and language exposure?   \| 1  (Strongly disagree) \| 2 \| 3 \| 4 \| 5  (Strongly agree) \| \| --- \| --- \| --- \| --- \| --- \|   **※ Words that posed difficulties in relation to age or generational context, such as 'radio,' were revised** \| \| **7)** Do you think the composition of word length (monosyllabic words, disyllabic words, and words with three or more syllables) is appropriate?  **※ Consensus among the experts was achieved in the first round** \| \| **8)** Is it composed of stimulating words that can capture children's interest?   \| 1  (Strongly disagree) \| 2 \| 3 \| 4 \| 5  (Strongly agree) \| \| --- \| --- \| --- \| --- \| --- \|   **※ Based on the consensus that the words should be highly familiar and clearly visualizable, they were replaced with alternative terms.** \|   **Section Ⅲ. Diversity and Necessity of Phonological Context Composition**   \| **1)** Do you think the current word list includes a balanced representation of target consonant positions (word-initial, word-medial, word-final), as well as various places and manners of articulation, and features (e.g., plosives, fricatives, labials)?   \| 1  (Strongly disagree) \| 2 \| 3 \| 4 \| 5  (Strongly agree) \| \| --- \| --- \| --- \| --- \| --- \|     **※ Based on expert recommendations, a distinction was made between medial and final coda consonants** \| \| --- \| --- \| --- \| --- \| --- \| --- \| \| **2)** Do you consider the selection of target vowels in the word list to be appropriate? In addition, do you think the newly added diphthongs are clinically useful?   \| 1  (Strongly disagree) \| 2 \| 3 \| 4 \| 5  (Strongly agree) \| \| --- \| --- \| --- \| --- \| --- \|     **※ Based on expert recommendations, additional words with diphthongs were included** \| \| **3)** Do you think the target phonological contexts presented in the table adequately reflect contexts in which errors are frequently observed in actual child speech production?   \| 1  (Strongly disagree) \| 2 \| 3 \| 4 \| 5  (Strongly agree) \| \| --- \| --- \| --- \| --- \| --- \|     **※ While suggestions were made to add sentence-level evaluations, the current assessment tool was maintained as word-based following consensus among the research team** \| \| **4)** Have words that could cause errors for speakers using dialects or regional variations been excluded?   \| 1  (Strongly disagree) \| 2 \| 3 \| 4 \| 5  (Strongly agree) \| \| --- \| --- \| --- \| --- \| --- \|   **※ Alternative words were added to avoid the potential dialectal variations of certain terms.** \| \| **5)** In the case of diphthongs, evaluating them together with consonants has the drawback of increasing difficulty and vocabulary load. Do you think it would be appropriate to provide an option to include additional assessments if deemed necessary (according to the therapist’s opinion)?   \| 1  (Strongly disagree) \| 2 \| 3 \| 4 \| 5  (Strongly agree) \| \| --- \| --- \| --- \| --- \| --- \| \|   **Section Ⅳ. Validity of Scoring Criteria and Feature-Based Assessment Method**   \| **1)** Do you clearly understand the distinctions among various indices for articulation accuracy (e.g., Percentage of Consonants Correct; PCC, Phonological Mean Length of Utterance; PMLU, Proportion of Whole-word Proximity; PWP, Percentage of Vowels Correct; PVC)?   \| 1  (Strongly disagree) \| 2 \| 3 \| 4 \| 5  (Strongly agree) \| \| --- \| --- \| --- \| --- \| --- \|   **※ Due to difficulties in understanding PWC and PWP, revisions were made to provide clearer guidelines and supplementary explanations** \| \| --- \| --- \| --- \| --- \| --- \| --- \| \| **2)** Do you think these indices help capture children’s articulation characteristics more precisely in clinical practice?   \| 1  (Not helpful at all) \| 2 \| 3 \| 4 \| 5  (Very helpful) \| \| --- \| --- \| --- \| --- \| --- \|   **※ An additional consensus round** \| \| **3)** Do you think the current classification of consonant error types (e.g., phonological substitution errors, assimilation errors) by detailed articulation features is valid as an assessment tool?   \| 1  (Not valid at all) \| 2 \| 3 \| 4 \| 5  (Very valid) \| \| --- \| --- \| --- \| --- \| --- \|   **※ An additional consensus round**  **※ Based on expert recommendations, an additional component was included to enable direct identification of the phoneme errors corresponding to each target phoneme** \| \| **4)** Do you think the classification criteria in the scoring table (place of articulation, manner of articulation, voicing, distinctive features, etc.) are clinically clear and distinguishable?   \| 1  (Very unclear) \| 2 \| 3 \| 4 \| 5  (Very clear) \| \| --- \| --- \| --- \| --- \| --- \|   **※ An additional consensus round**  **※ There were conflicting recommendations: one suggested classification by place of articulation, manner of articulation, phonation type, and distinctive features, with the latter further subdivided into place, manner, and phonation type features; while another recommended simplification. Through internal expert consensus, a compromise was reached and revisions were made accordingly** \| \| **5)** Do you think the current scoring calculation method (e.g., denominator based on total number of items, ratio representation) is appropriate for respondents or clinicians to understand and apply?   \| 1  (Very inappropriate) \| 2 \| 3 \| 4 \| 5  (Very appropriate) \| \| --- \| --- \| --- \| --- \| --- \|   **※ An additional consensus round**  **※ The numbers in the denominators were revised on the basis of the survey** \| \| **6)** Do the current scoring indices (e.g., PCC, PMLU, PWP, PVC) provide sufficient information for clinically interpreting children’s articulation and phonological abilities?   \| 1  (Strongly disagree) \| 2 \| 3 \| 4 \| 5  (Strongly agree) \| \| --- \| --- \| --- \| --- \| --- \|     **※ An additional consensus round**  **※ As the interpretation of PWC and PWP was difficult, an additional consensus round will be conducted** \| \| **7)** Do you think the classifications in the current scoring criteria (features, place of articulation, manner of articulation, error types, etc.) are overly subdivided or contain redundancies?   \| 1  (Strongly disagree) \| 2 \| 3 \| 4 \| 5  (Strongly agree) \| \| --- \| --- \| --- \| --- \| --- \|     **※ Overly detailed and redundant items were streamlined** \| \| **8)** Do you think this scoring index system is appropriate for explaining changes in articulation abilities before and after treatment to parents or caregivers?   \| 1  (Strongly disagree) \| 2 \| 3 \| 4 \| 5  (Strongly agree) \| \| --- \| --- \| --- \| --- \| --- \|   **※ Consensus among the experts was achieved in the first round**  **※ Graphs of the proportion for each index will be presented, and where normative data exist, the child’s accuracy will be visualized in comparison to these norms** \| \| **9)** If you believe there are aspects that should be improved to increase the discriminant validity of the assessment, please describe them.  **※ Additional evaluations of diphthongs and diverse coda consonants were added as recommended** \| \| **10)** Do you think using PWC and PWP analyses is helpful in setting actual treatment goals?   \| 1  (Strongly disagree) \| 2 \| 3 \| 4 \| 5  (Strongly agree) \| \| --- \| --- \| --- \| --- \| --- \|     **※ Modifications were made in accordance with expert opinions** \| \| **11)** Do you think the PMLU scale effectively contributes to evaluating phonological complexity in children?   \| 1  (Strongly disagree) \| 2 \| 3 \| 4 \| 5  (Strongly agree) \| \| --- \| --- \| --- \| --- \| --- \|     **※ Modifications were made in accordance with expert opinions** \| \| **12)** Do you think providing accuracy analysis results by manner of articulation (plosives, fricatives, etc.) and place of articulation (bilabials, velars, etc.) is useful for clinical interpretation?   \| 1  (Strongly disagree) \| 2 \| 3 \| 4 \| 5  (Strongly agree) \| \| --- \| --- \| --- \| --- \| --- \|   **※ Consensus among the experts was achieved in the first round** \| \| **13)** Do you think introducing assessment indices based on distinctive features (continuancy, stridency, anteriority, etc.) can meaningfully contribute to error pattern analysis?   \| 1  (Strongly disagree) \| 2 \| 3 \| 4 \| 5  (Strongly agree) \| \| --- \| --- \| --- \| --- \| --- \|   **※ Consensus among the experts was achieved in the first round** \| \| **14)** Do you think it is necessary to provide a comprehensive report format that includes multiple analysis indices (PCC, PWC, feature-based indices, etc.)?   \| 1  (Strongly disagree) \| 2 \| 3 \| 4 \| 5  (Strongly agree) \| \| --- \| --- \| --- \| --- \| --- \|   **※ Consensus among the experts was achieved in the first round** \| |
| --- | --- | --- | --- | --- | --- | --- | --- | --- | --- | --- | --- | --- | --- | --- | --- | --- | --- | --- | --- | --- | --- | --- | --- | --- | --- | --- | --- | --- | --- | --- | --- | --- | --- | --- | --- | --- | --- | --- | --- | --- | --- | --- | --- | --- | --- | --- | --- | --- | --- | --- | --- | --- | --- | --- | --- | --- | --- | --- | --- | --- | --- | --- | --- | --- | --- | --- | --- | --- | --- | --- | --- | --- | --- | --- | --- | --- | --- | --- | --- | --- | --- | --- | --- | --- | --- | --- | --- | --- | --- | --- | --- | --- | --- | --- | --- | --- | --- | --- | --- | --- | --- | --- | --- | --- | --- | --- | --- | --- | --- | --- | --- | --- | --- | --- | --- | --- | --- | --- | --- | --- | --- | --- | --- | --- | --- | --- | --- | --- | --- | --- | --- | --- | --- | --- | --- | --- | --- | --- | --- | --- | --- | --- | --- | --- | --- | --- | --- |

**Supplementary Table 3.** Round 3 Delphi questionnaire

| **Section Ⅱ. Appropriateness of Word List and Stimulus Word Composition**   \| **1)** Do you think the current word list included in the articulation assessment tool aligns with the purpose of the assessment (e.g., phoneme coverage, diversity of articulation contexts)?  **※ Consensus among the experts was achieved in the second round** \| \| --- \| \| **2)** If there are articulation contexts that you believe are missing from the presented word list (e.g., voiced fricatives, complex final consonants), please specify them.  **※ No additional comments** \| \| **3)** Do you think the current word list is appropriate for covering children aged 2 to 18 years, and that it is composed of vocabulary familiar to children in clinical settings?  **※ Consensus among the experts was achieved in the second round** \| \| **4)** In your opinion, is the revised word list suitable for visualization and readily comprehensible to children?  **※ Consensus among the experts was achieved in the second round** \| \| **5)** Do you think the order in which the words are presented is appropriate in terms of assessment flow or difficulty level?  **※ Consensus among the experts was achieved in the second round** \| \| **6)** Do you think the word list is appropriately composed considering age-appropriate word frequency and language exposure?  **※ Consensus among the experts was achieved in the second round** \| \| **7)** Do you think the composition of word length (monosyllabic words, disyllabic words, and words with three or more syllables) is appropriate?  **※ Consensus among the experts was achieved in the first round** \| \| **8)** Is it composed of stimulating words that can capture children's interest?  **※ Consensus among the experts was achieved in the second round** \|   **Section Ⅲ. Diversity and Necessity of Phonological Context Composition**   \| **1)** Do you think the current word list includes a balanced representation of target consonant positions (word-initial, word-medial, word-final), as well as various places and manners of articulation, and features (e.g., plosives, fricatives, labials)?  **※ Consensus among the experts was achieved in the second round** \| \| --- \| \| **2)** Do you consider the selection of target vowels in the word list to be appropriate? In addition, do you think the newly added diphthongs are clinically useful?  **※ Consensus among the experts was achieved in the second round** \| \| **3)** Do you think the target phonological contexts presented in the table adequately reflect contexts in which errors are frequently observed in actual child speech production?   \| 1  (Strongly disagree) \| 2 \| 3 \| 4 \| 5  (Strongly agree) \| \| --- \| --- \| --- \| --- \| --- \|   **※ An additional consensus round** \| \| **4)** Have words that could cause errors for speakers using dialects or regional variations been excluded?  **※ Consensus among the experts was achieved in the second round** \| \| **5)** In the case of diphthongs, evaluating them together with consonants has the drawback of increasing difficulty and vocabulary load. Do you think it would be appropriate to provide an option to include additional assessments if deemed necessary (according to the therapist’s opinion)?  **※ No additional comments** \|   **Section Ⅳ. Validity of Scoring Criteria and Feature-Based Assessment Method**   \| **1)** Do you clearly understand the distinctions among various indices for articulation accuracy (e.g., Percentage of Consonants Correct; PCC, Phonological Mean Length of Utterance; PMLU, Proportion of Whole-word Proximity; PWP, Percentage of Vowels Correct; PVC)?  **※ Consensus among the experts was achieved in the second round** \| \| --- \| \| **2)** Do you think these indices help capture children’s articulation characteristics more precisely in clinical practice?  **※ Consensus among the experts was achieved in the second round** \| \| **3)** Do you think the current classification of consonant error types (e.g., phonological substitution errors, assimilation errors) by detailed articulation features is valid as an assessment tool?  **※ Consensus among the experts was achieved in the second round** \| \| **4)** Do you think the classification criteria in the scoring table (place of articulation, manner of articulation, voicing, distinctive features, etc.) are clinically clear and distinguishable?  **※ Consensus among the experts was achieved in the second round** \| \| **5)** Do you think the current scoring calculation method (e.g., denominator based on total number of items, ratio representation) is appropriate for respondents or clinicians to understand and apply?  **※ Consensus among the experts was achieved in the second round** \| \| **6)** Do the current scoring indices (e.g., PCC, PMLU, PWP, PVC) provide sufficient information for clinically interpreting children’s articulation and phonological abilities?  **※ Consensus among the experts was achieved in the second round** \| \| **7)** Does the current scoring rubric appear to be overly detailed or contain redundancies?   \| 1  (Strongly disagree) \| 2 \| 3 \| 4 \| 5  (Strongly agree) \| \| --- \| --- \| --- \| --- \| --- \|   **※ The Percent Word Proximity (PWP) index at the word level was excluded from the assessment** \| \| **8)** Do you think this scoring index system is appropriate for explaining changes in articulation abilities before and after treatment to parents or caregivers?  **※ Consensus among the experts was achieved in the second round** \| \| **9)** If you believe there are aspects that should be improved to increase the discriminant validity of the assessment, please describe them.  **※ No additional comments** \| \| **10)** Does the use of PWC in analysis contribute to establishing practical therapy goals?   \| 1  (Strongly disagree) \| 2 \| 3 \| 4 \| 5  (Strongly agree) \| \| --- \| --- \| --- \| --- \| --- \|   **※ The Percent Word Proximity (PWP) index at the word level was excluded from the assessment** \| \| **11)** Would incorporating an additional measurement of the Phonological Mean Length of Utterance (PMLU) provide supplementary support to the evaluation outcomes?   \| 1  (Strongly disagree) \| 2 \| 3 \| 4 \| 5  (Strongly agree) \| \| --- \| --- \| --- \| --- \| --- \|   **※ An additional consensus round** \| \| **12)** Do you think providing accuracy analysis results by manner of articulation (plosives, fricatives, etc.) and place of articulation (bilabials, velars, etc.) is useful for clinical interpretation?  **※ Consensus among the experts was achieved in the second round** \| \| **13)** Would the inclusion of consonant classification features in the additional evaluation contribute to the analysis of error patterns?   \| 1  (Strongly disagree) \| 2 \| 3 \| 4 \| 5  (Strongly agree) \| \| --- \| --- \| --- \| --- \| --- \|   **※ An additional consensus round** \| \| **14)** Do you think it is necessary to provide a comprehensive report format that includes multiple analysis indices (PCC, PWC, feature-based indices, etc.)?  **※ Consensus among the experts was achieved in the second round** \| |
| --- | --- | --- | --- | --- | --- | --- | --- | --- | --- | --- | --- | --- | --- | --- | --- | --- | --- | --- | --- | --- | --- | --- | --- | --- | --- | --- | --- | --- | --- | --- | --- | --- | --- | --- | --- | --- | --- | --- | --- | --- | --- | --- | --- | --- | --- | --- | --- | --- | --- | --- | --- | --- |
